# Quantitative and Qualitative Deficits in Neonatal Lung-Migratory Dendritic Cells Impact the Generation of the CD8+ T Cell Response

**DOI:** 10.1371/journal.ppat.1003934

**Published:** 2014-02-13

**Authors:** Tracy J. Ruckwardt, Allison M. W. Malloy, Kaitlyn M. Morabito, Barney S. Graham

**Affiliations:** Vaccine Research Center, National Institute of Allergy and Infectious Disease, National Institutes of Health, Bethesda, Maryland, United States of America; Mount Sinai School of Medicine, United States of America

## Abstract

CD103+ and CD11b+ populations of CD11c+MHCIIhi murine dendritic cells (DCs) have been shown to carry antigens from the lung through the afferent lymphatics to mediastinal lymph nodes (MLN). We compared the responses of these two DC populations in neonatal and adult mice following intranasal infection with respiratory syncytial virus. The response in neonates was dominated by functionally-limited CD103+ DCs, while CD11b+ DCs were diminished in both number and function compared to adults. Infecting mice at intervals through the first three weeks of life revealed an evolution in DC phenotype and function during early life. Using TCR transgenic T cells with two different specificities to measure the ability of CD103+ DC to induce epitope-specific CD8+ T cell responses, we found that neonatal CD103+ DCs stimulate proliferation in a pattern distinct from adult CD103+ DCs. Blocking CD28-mediated costimulatory signals during adult infection demonstrated that signals from this costimulatory pathway influence the hierarchy of the CD8+ T cell response to RSV, suggesting that limited costimulation provided by neonatal CD103+ DCs is one mechanism whereby neonates generate a distinct CD8+ T cell response from that of adults.

## Author Summary

Respiratory syncytial virus (RSV) infection is most severe in infants under six months and the most common cause of hospitalization for lower respiratory tract infection in children under five years. Disease is a consequence of virus- and T-cell-mediated pathology. Adaptive immune responses to viral respiratory infections are initiated by dendritic cells (DCs) that traffic to lymph nodes from the infected lungs. We compared the phenotype and function of two lung-migratory DC populations, identified by high expression of MHC Class II and CD103+ (CD103+DCs) or CD11b+ (CD11b+DCs), in mice infected in early life or as adults. We found that DC subsets undergo dramatic quantitative and qualitative changes during the first weeks of life, and CD103+DCs from neonatal mediastinal lymph nodes induced a fundamentally different CD8+ T-cell response profile than CD103+DCs from adults. The adult response pattern required CD28-mediated costimulatory signals, which is a limiting functional property of neonatal CD103+DCs. Thus, the ability of neonatal CD103+DCs to provide sufficient costimulation to neonatal CD8+ T-cells can influence the immunodominance hierarchy and functional properties of neonatal CD8+ T-cell responses compared to those of adults. A better understanding of deficiencies in early life immunity will guide vaccine approaches that induce disease-sparing immune responses in infants.

## Introduction

Four major populations of dendritic cells have been defined in lung [1]–[3]. Plasmacytoid DCs (pDCs) and two migratory dendritic cell populations, distinguished by the expression of either CD103 or CD11b, are responsible for lung surveillance in the steady-state and are poised to detect and respond rapidly to environmental and microbial threats. Under inflammatory conditions, monocyte-derived DCs are recruited, offering further support for the local immune response. Recent studies have revealed specialization of these subsets, and a critical role for the CD103+ and CD11b+ tissue-resident populations of DCs in the induction of the adaptive response. CD103+ DCs, primarily located in the basal lamina, and CD11b+ DCs, situated in the lamina propria, are the two populations capable of capturing antigen and migrating through afferent lymphatics to mediastinal lymph nodes (MLN) to initiate and orchestrate adaptive immune responses. CD103+ DCs, and the related CD8α+ lymph node-resident DC subset, readily cross-present antigens [4], [5], and CD103+ DCs have been shown to potently induce CD8+ T cell responses [6]–[11]. Batf3-deficient mice, lacking both tissue-resident CD103+ DCs and CD8α+ DCs, show a marked reduction in CD8+ T cell responses [12], [13], and CD103+ DCs have also been shown to have the unique capability to transport apoptotic cells to the MLN [14]. While the role of CD103+ DCs in the induction of CD4+ T cell-mediated immunity is a matter of debate, CD103+ DCs have been found to induce CD4+ T cell responses in several studies, sometimes eliciting distinct effector profiles than the CD11b+ population [7], [9], [15], [16]. CD11b+ DCs produce chemokines in the lung [17], and following migration to the MLN, have a clear role in the stimulation of CD4+ responses. Recently, CD11b+ DCs have also been shown to mediate and maintain allergic airway sensitization [18], [19]. The anatomical location of CD103+ and CD11b+ DCs, combined with their migratory capabilities and potent ability to induce adaptive responses in the lymph node make them critical mediators of the immune response to virus infections of the respiratory tract. The composition and function of these two types of dendritic cell in the MLN is likely to dictate the outcome of adaptive immune responses.

Early life is known to be associated with increased susceptibility to infections. This vulnerability is related to both the immaturity and inexperience of the neonatal/infant immune response. Lower respiratory tract infections (LRTI), in particular, have a large impact on early life mortality. Globally, it is estimated that LRTI are responsible for a significant portion of neonatal (0–27 days, 6.8%), post-neonatal (28–364 days, 20.1%), and child (1–4 years, 12.4%) deaths [20]. These estimates of mortality underlie the large health and economic burden of respiratory infections in the young, where hospitalization and supportive care is often a requirement for recovery. Respiratory syncytial virus (RSV) is the most important viral pathogen in early life and accounts for the largest fraction of deaths due to LRTI in the first year [20]. Vaccination during the first few weeks of life would be the most effective approach to mitigating the morbidity and mortality of severe RSV disease, but is complicated by the hyporesponsiveness of infant immune responses.

Despite the increased susceptibility to respiratory infection in early life, and the described shortcomings of early life immunity, little is known about how lung dendritic cells function in early life and how deficits in the DC response affect the outcome of adaptive immunity. We sought to define the dynamics of early-life lung-migratory dendritic cell populations following viral infection with RSV. We focused on CD103+ and CD11b+ DC populations in the MLN following infection of mice, and compared their phenotype and function in early life to those of adults. We found evidence for direct infection of both DC populations, indicating the potential for both direct and cross-presentation of antigen in the MLN. Infecting mice in the first three weeks of life revealed an early-life evolution in DC phenotype and function. Neonatal CD11b+ responses were 10-fold lower during early life and demonstrated functional deficits in the uptake and processing of soluble antigen. CD103+ DCs comprised the majority of the neonatal response and were quantitatively similar to those of adults but exhibited deficiencies in the uptake and processing of soluble antigen as well as in upregulation of costimulatory ligands. Neonatal CD103+ DCs induced proliferation in two RSV-specific CD8+ T cell populations in a distinctly different pattern than adult CD103+ DCs, indicating an age-dependent difference in their ability to process and present antigens or to provide co-stimulatory signals that may impact the generation and immunodominance profile of the CD8+ T cell response following virus infection. By recapitulating conditions of limited CD28-mediated costimulation during adult infection, we provide evidence that signaling through CD28 differentially impacts epitope-specific CD8+ T cell responses and that reduced expression of CD86 and CD80 constitutes one mechanism by which neonatal mice establish an epitope hierarchy that is distinct from that of adults.

## Results

### RSV directly infects murine respiratory dendritic cells

**Figure 1:**
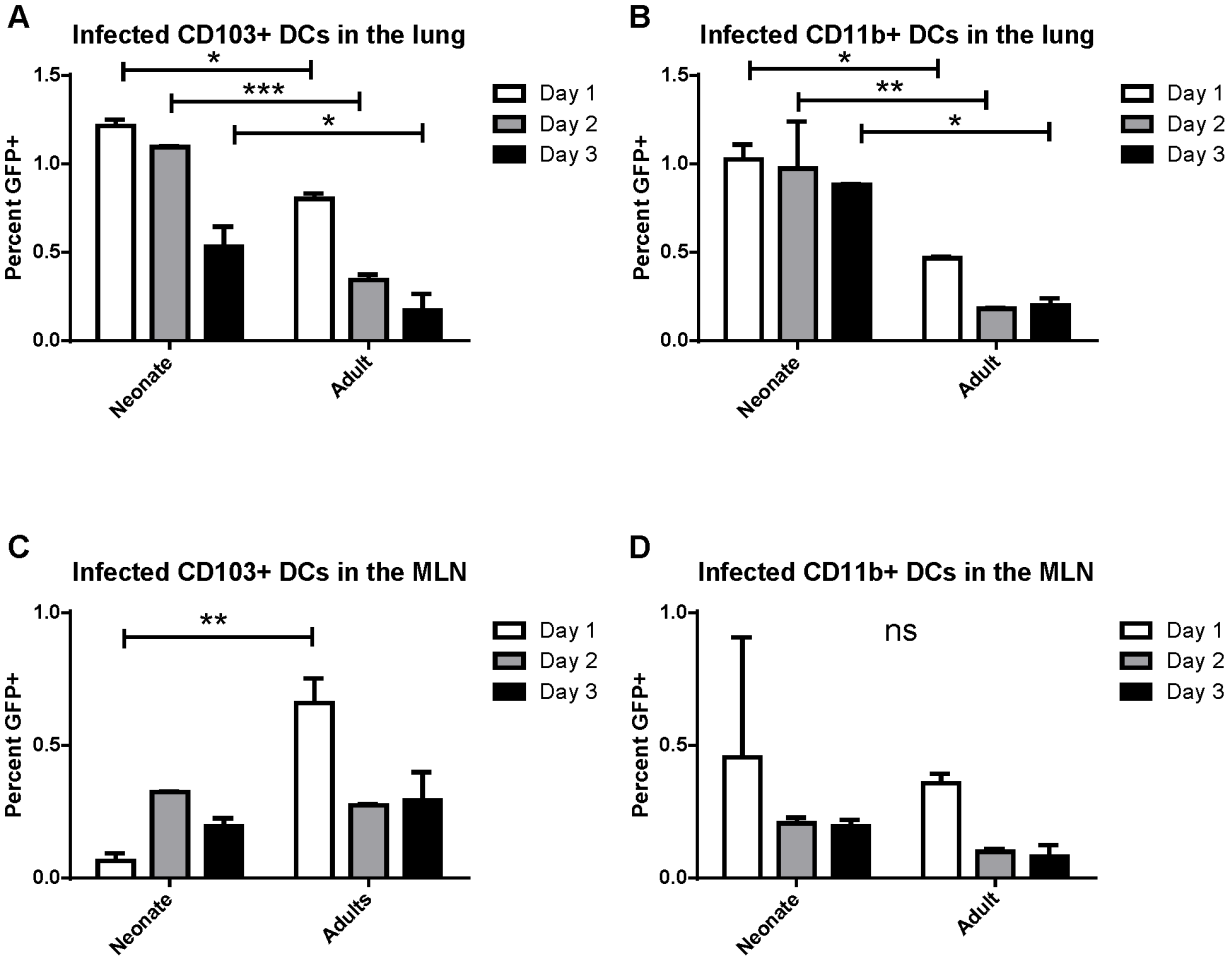
RSV infection of lung migratory dendritic cell populations in the lung and draining mediastinal lymph node. Percent of infected (GFP+) dendritic cell populations in the lung (A and B) and the posterior mediastinal lymph node (MLN, C and D) at days 1–3 post-infection. Lung and MLN DC populations were gated as described in Figure S1 and S2, respectively. Percent GFP+ of CD103+ DCs (A and C) and CD11b+ DCs (B and D) was determined by comparison to mice infected at the same time with wtRSV. Data is representative of two experiments done with two pooled samples of 3–7 mice each and the error bars represent the SEM. * p≤0.05, **p≤0.01, ***p≤0.001, ns is no significance by two-way ANOVA and Sidak's multiple comparisons test.

Previously, real-time RT-PCR for the N gene was used to demonstrate the presence of RSV viral RNA in sorted CD103+ and CD11b+ dendritic cell subsets that had migrated to the draining mediastinal lymph node (MLN) following RSV infection [21]. These sorted dendritic cells were able to present RSV antigen to both CD4+ and CD8+ T cells as measured by IFNγ production by co-cultured RSV-specific T cells. Because these results may reflect uptake of cell debris from infected lungs by dendritic cells and cross-presentation of antigen rather than direct infection, we sought to determine whether RSV directly infects CD103+ and CD11b+ populations of lung-migratory dendritic cells following primary acute RSV infection. As a more reliable measure of direct infection, adult and neonatal (7 day old) mice were infected with either 2×10^6^ PFU of wild-type (wt) or recombinant green (rg) RSV at day 0 and lungs and mediastinal lymph nodes were harvested at 1, 2, and 3 days post-infection. As GFP is expressed only by cells directly infected with rgRSV, CD103+ and CD11b+ cells were assessed for GFP expression and compared to control infection with wtRSV (gating strategy presented in Figure S1, representative data demonstrating GFP expression in CD103+ DCs in the lungs of rgRSV-infected neonates and adults are shown in Figure S2). In the lungs of rgRSV infected mice, GFP expression in both CD103+ (Figure 1A) and CD11b+ (Figure 1B) DCs peaked at one day post-infection with approximately 1% of dendritic cells being directly infected. Neonates showed a higher frequency of directly infected CD103+ and CD11b+ than adult mice. This result was not surprising given our previous finding that neonatal mice have a higher viral titer in the lungs early in infection than adult mice [22]. Neonates also showed less of a decline in the number of infected dendritic cells in the lung than adult mice in the first three days following infection, which may indicate a delay in their migration to the MLN.

The posterior mediastinal lymph node is the inductive site for the adaptive immune response against lung pathogens in mice. We asked if infected dendritic cells migrated and could be found in the MLN between 1 and 3 days post-infection (pictures of the mediastinal lymph node in neonates and adults and the gating strategy for lymph node analysis is presented in Figure S3). Again, the percentage of CD103+ and CD11b+ cells that were GFP+ following rgRSV infection was determined by comparison with wtRSV infection. We found that direct infection of CD103+ dendritic cells peaked in adults at 1 day post infection, while infected CD103+ DCs peaked in neonates at day 2 post-infection (Figure 1C). Infection of CD11b+ DCs peaked in both neonates and adults at one day post-infection (Figure 1D). These data demonstrate for the first time that both lung-migratory CD103+ and CD11b+ DC populations can be directly infected with RSV, albeit at a low frequency, and can be detected in MLN.

### The response of lung-migratory dendritic cells evolves dramatically during early life

**Figure 2:**
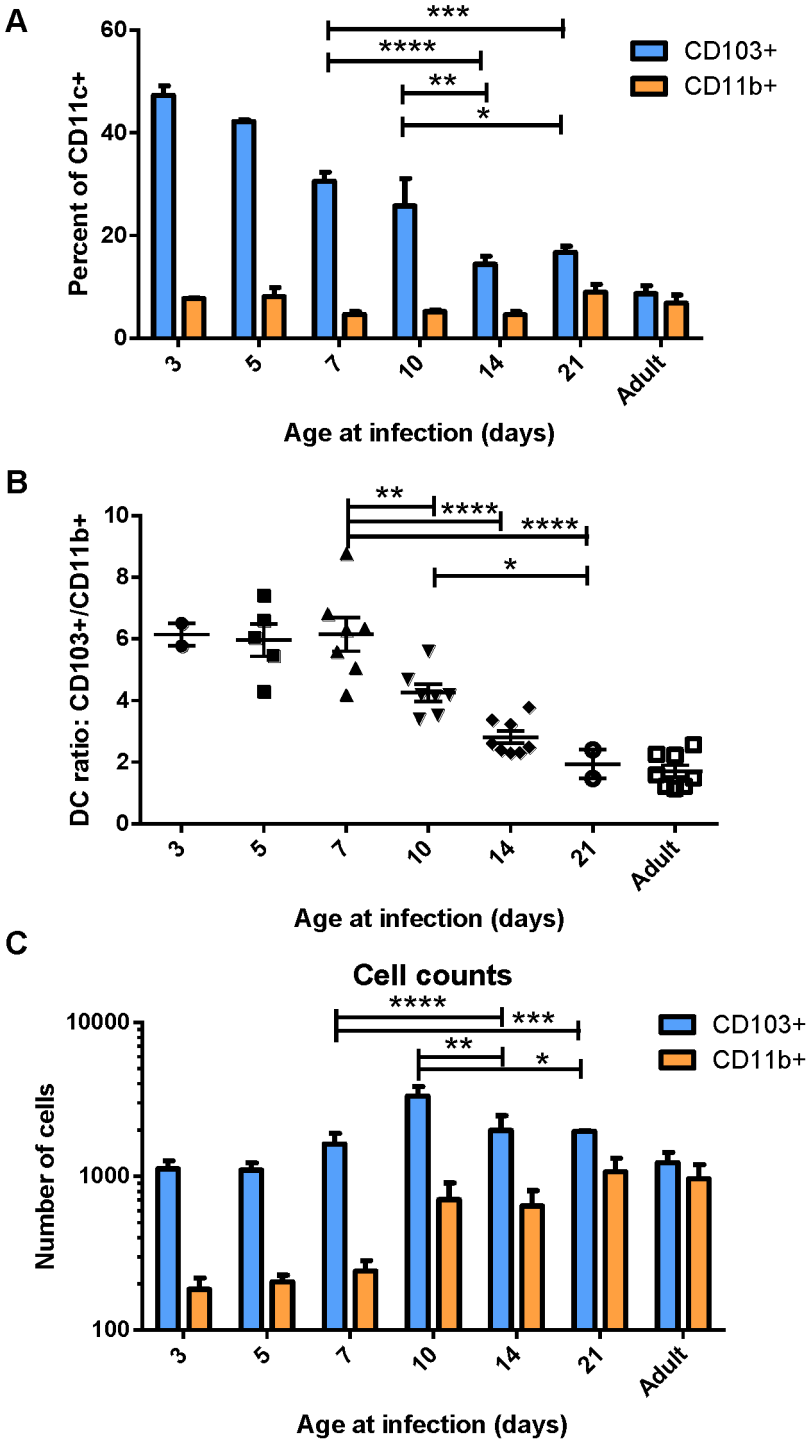
Lung-migratory dendritic cell responses change dramatically during early life. CD103+ and CD11b+ DC populations in the MLN three days post-RSV infection were measured in mice of different ages. A) DC populations as a percent of CD11c+-gated cells of mice infected at the indicated age. B) Ratio of CD103+ to CD11b+ cells in the MHC Class II high gate. C) Total number of DC cells acquired on the flow cytometer per mouse after running samples to completion. Data in A and C are representative of three experiments using mice at available ages, and B represents data compiled from three independent experiments. Groups were compared using a two-way or one-way ANOVA and Tukey's multiple comparisons tests in GraphPad Prism and significant differences within the 7–21 day transition period are indicated (* p≤0.05, ** p≤0.01, ***p≤0.001, **** p≤0.0001). Error bars represent the SEM.

Because DCs in the MLN are responsible for eliciting the adaptive response to RSV following infection, we focused on the characterization of DC populations in the MLN following infection of mice at different ages. We infected mice between 3 days old and 21 days old, and compared their DC responses to those of adults 3 days post-infection. Strikingly, we found that the DC response in young mice was predominantly composed of CD103+ DCs (Figure 2A). This was particularly evident in the youngest mice infected, where nearly 50% of all CD11c+ DCs in the MLN were CD103+. Mice infected between 3 days old and 21 days old showed a rapid evolution in the composition of DCs in the MLN (representative flow cytometry plots are shown in Figure S4). Mice infected at 3 days old had 6-fold more CD103+ DCs in the MLN than CD11b+ DCs (Figure 2B). The ratio of CD103+ to CD11b+ DCs (frequency of CD103+/frequency of CD11b+) remained at 6 in mice infected at or before 7 days old. Mice infected at 10, 14, and 21 days old showed a progressive shift from a 6-fold ratio to the 1.5 to 2-fold ratio found in infected adult mice (Figure 2B). We quantified the absolute number of CD103+ and CD11b+ present in the MLN by acquiring samples to completion by flow cytometry. Plotting absolute numbers showed that neonates and adults had similar numbers of CD103+ DCs in the LN at three days post-infection, while CD11b+ DCs in neonates were approximately 10-fold lower in mice infected between three and seven days of life. Again, mice infected between 10 and 21 days of life showed a progressive increase in CD11b+ DCs, with 21 day old mice showing similar numbers of both CD103+ and CD11b+ DCs in the MLN as infected adult mice (Figure 2C). We observed a similar CD103-biased composition of naïve neonatal (7 day old) MLNs as compared to those of an adult, suggesting that a CD103-dominated response may be a general feature of the neonatal response to respiratory pathogens (Figure S5).

### Early-life lung migratory dendritic cells display deficits in both transport and processing of soluble antigen

**Figure 3:**
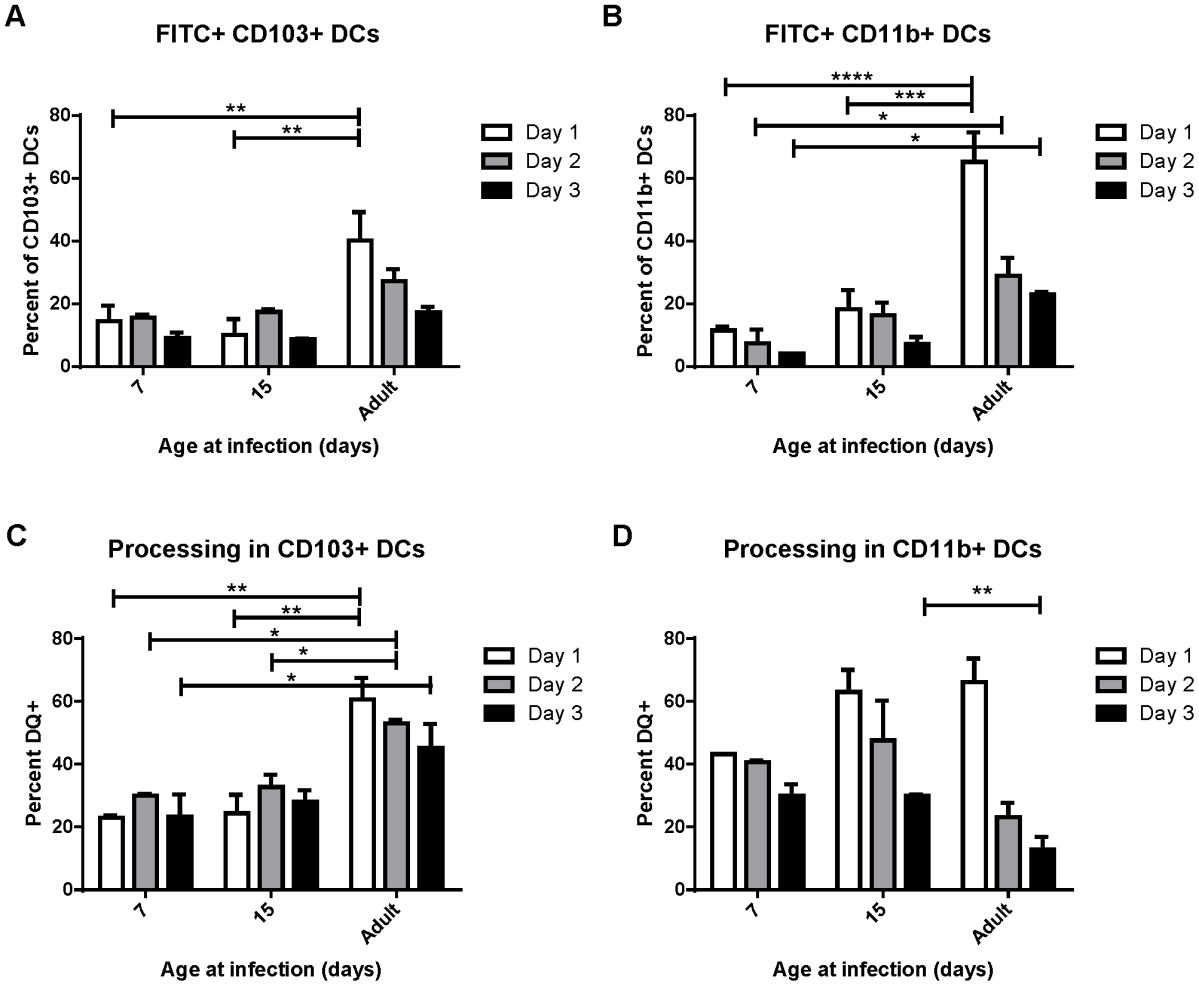
Uptake and processing of soluble antigen is age-dependent. Soluble ovalbumin-FITC protein (A and B) or ovalbumin-DQ (C and D) were co-administered at the time of RSV infection and the percent of the CD103+ and CD11b+ DC populations positive for FITC expression (A or B) or shown to be processing antigen by unquenching of ova-DQ were measured in the MLN at days 1, 2, and 3 post-infection. Data are combined from two independent experiments using pooled lymph node samples (3–7 mice/pool depending on age and availability) and error bars indicate the SEM. * p≤0.05, **p≤0.01, ***p≤0.001, **** p≤0.0001 by two-way ANOVA and Tukey's multiple comparisons test.

Uptake, transport and processing of soluble antigens are critical functions of lung-migratory dendritic cells. We assessed the ability of DCs from mice of different ages to both transport and process soluble antigen (Figure 3). Dendritic cell transport of soluble antigen was measured by co-administration of 50 µg of FITC-labeled ovalbumin at the time of RSV infection. Percentages of DCs expressing FITC were measured in the MLN at 1, 2, and 3 days post-infection (representative data showing FITC+ CD103+ DCs in the lymph node at each age is shown in Figure S6A). Both CD103+ and CD11b+ DCs from mice infected at 7 and 15 days old displayed a reduced ability to transport soluble antigen to the lymph node than those from adults (Figure 3, A and B). Additionally, FITC+ DCs peaked in adult mice at day 1 post-infection, and tended to peak at day 2 in mice infected in the first two weeks of life. Antigen processing was measured by co-instillation of DQ ovalbumin (DQ-ova). DQ-ova is a self-quenched conjugate that fluoresces upon proteolytic degradation, a requisite for presentation of MHC/peptide and the induction of adaptive T cell responses. Again, a lower frequency of CD103+ and CD11b+ DCs from mice infected in early life were positive, indicating that they had taken up and processed DQ-ova (Figure 3, C and D, representative data showing DQ+ CD103+ DCs in the lymph node at each age is shown in Figure S6B). As we described for uptake and transport of soluble antigen, neonatal CD103+ also demonstrated a one day delay in the peak of processing as compared to adult CD103+ (Figure 3C). The percentage of CD11b+ DCs processing antigen in neonates was more similar to that of adults, with no significant age-dependent difference in the frequency of cells processing antigen on day 1, the peak for mice infected at every age (Figure 3D). Interestingly, measurements of both uptake and processing of soluble antigen revealed that mice infected at 15 days had little, if any, improvement over mice infected at 7 days of age despite the changes we observed in DC composition in the MLN over this period of time (Figure 2).

### Early life lung-migratory dendritic cell populations have limited expression of costimulatory molecules

**Figure 4:**
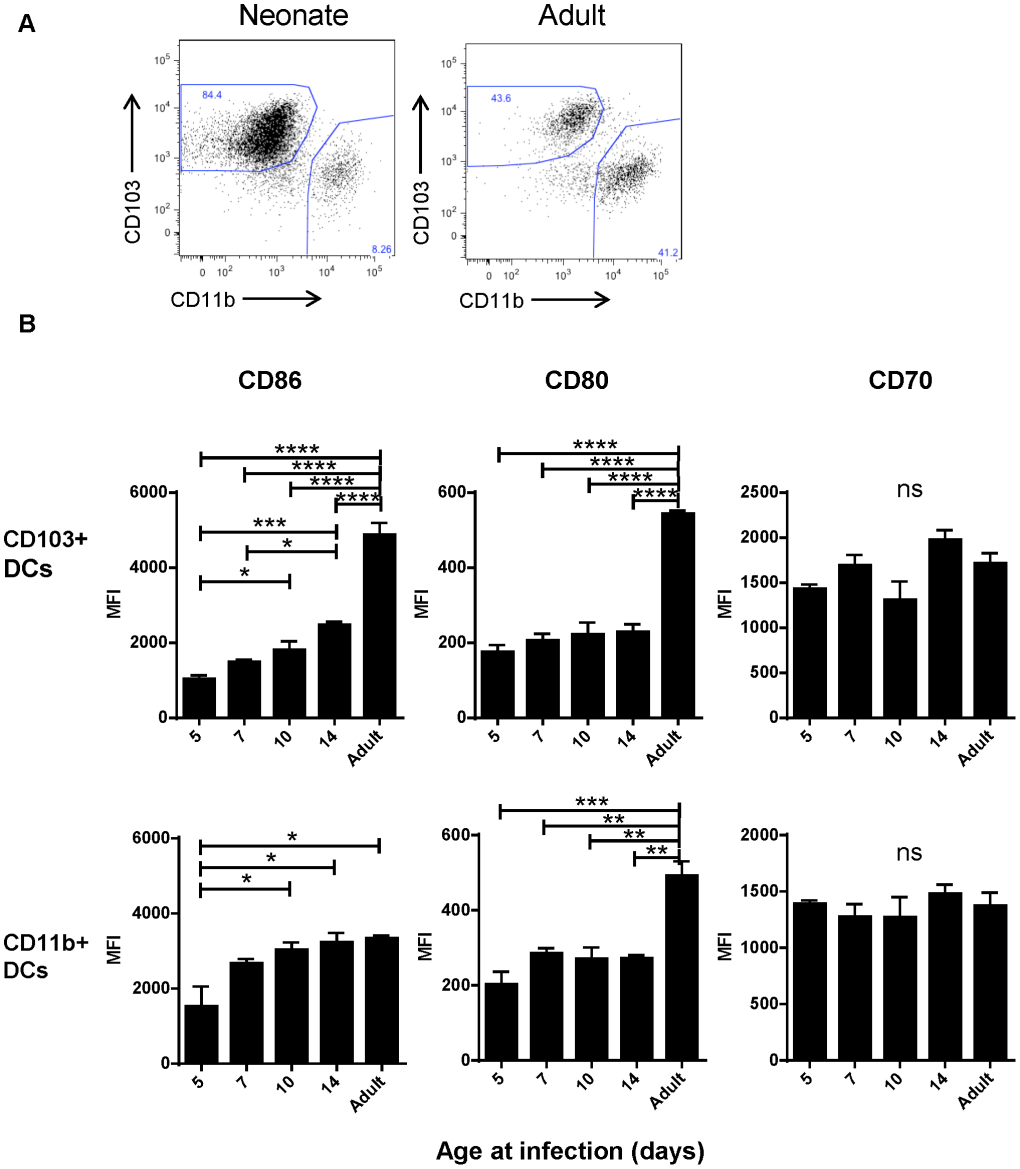
Age-dependent phenotypic changes in the CD103+ and CD11b+ DC populations in the MLN three days post-infection. A) Evaluation of the Class II high populations of neonatal (7 days old) vs. adult mice shows differences in composition and phenotype of DC populations. B) Costimulatory molecule (CD86, CD80 and CD70) expression was measured by flow cytometry on CD103+ and CD11b+ DCs three days post-infection at the indicated age and the mean fluorescence intensity (MFI) is displayed. Data are representative of three experiments using two to three samples of lymph nodes pooled from 3–7 mice. Error bars indicate the SEM, and all data was analyzed using one-way ANOVA with Tukey's multiple comparisons test (* p≤0.05, ** p≤0.01, *** p≤0.001, **** p≤0.0001).

Despite appearing in similar numbers, we observed that CD103+ DCs isolated from infected neonatal (7 day old) mice had lower expression of CD103+ than their adult counterparts (Figure 4A). We asked about the age-dependent expression of the costimulatory molecules CD86, CD80 and CD70 on the surface of CD103+ and CD11b+ DCs in the MLN at three days post-infection with RSV as an indication of their capacity to effectively induce adaptive responses. We found the most striking differences in the CD103+ population, which showed an age-dependent ability to upregulate both CD86 and CD80 expression following infection (Figure 4B, representative flow plots are shown in Figure S7). CD86 showed a pattern of significantly increasing expression with age at infection, and CD80 showed lower expression in mice infected at or under two weeks old than during adult infection. Age-dependent differences in CD86 expression on CD11b+ DCs were less pronounced, with the differences in expression being significant only between mice infected at 5 days old and older groups. The age-dependent pattern of CD80 expression on CD11b+ DCs was similar to that of CD103+ DCs. CD70 expression on both dendritic cell types was similar with no significant differences between mice infected at different ages (Figure 4B).

### Early life deficiencies in lung-migratory dendritic cells impact the adaptive immune response

**Figure 5:**
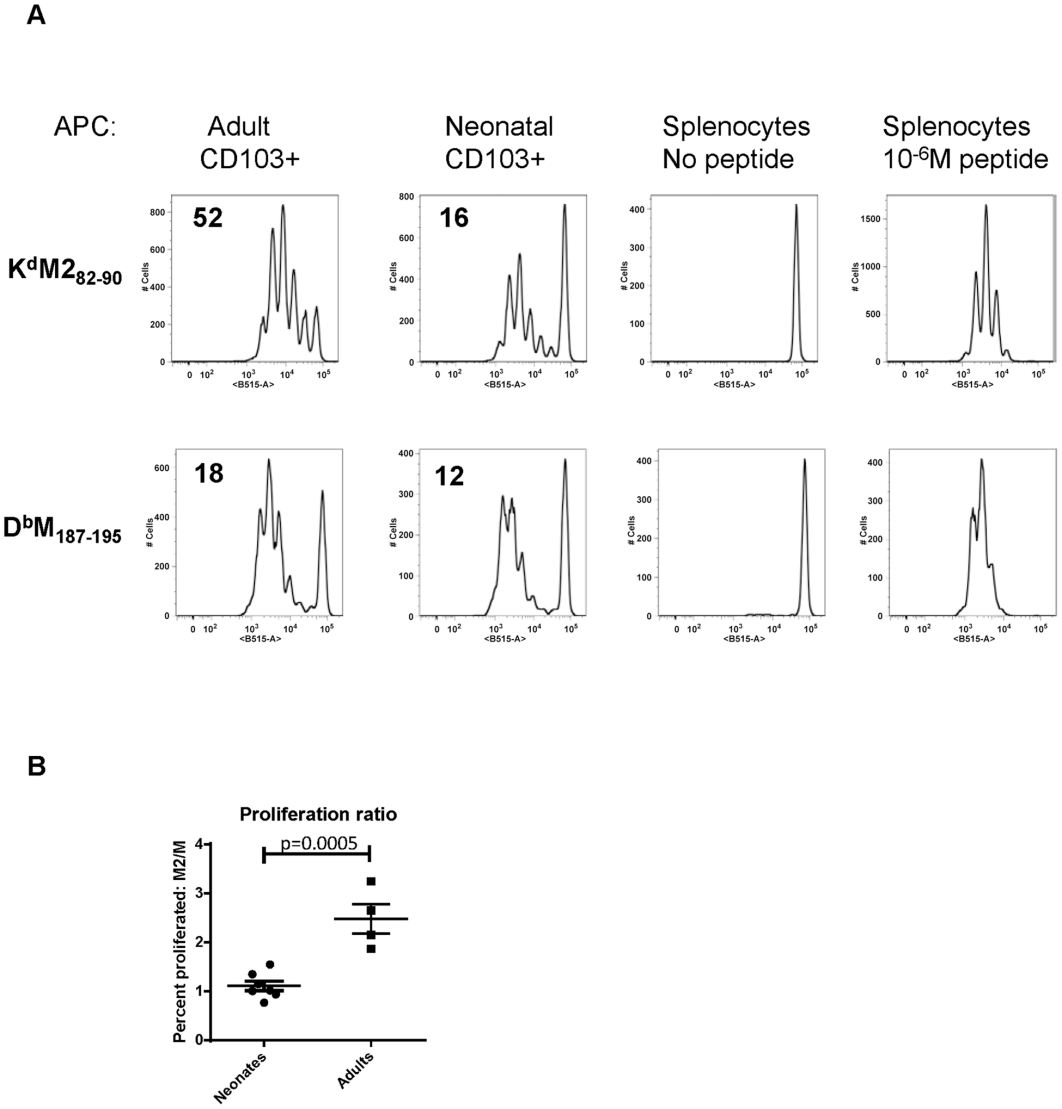
Neonatal CD103+ DCs differentially induce RSV-specific adaptive CD8+ T cell responses. A) CFSE-labeled TCR transgenic CD8+ T cells specific for K^d^M2_82–90_ or D^b^M_187–195_ were co-cultured in a 10∶1 ratio with CD103+ dendritic cells sorted from the lymph nodes of RSV infected adults or neonates one day after infection. The percent of cells that proliferated following three days in co-culture was calculated using the proliferation module in FlowJo 9.4.10. Negative control samples consisted of TCR Tg T cells co-cultured with splenocytes with no exogenous peptide, and positive control samples were co-cultured with splenocytes and 10^−6^M of specific peptide. B) Comparison of the ratio of proliferation (percent of K^d^M2_82–90_-specific cells that proliferated/percent of D^b^M_187–195_-specific cells that proliferated) induced by flow-sorted neonatal vs. adult CD103+DCs. Groups were compared using a student's t-test.

We have previously defined age-dependent differences in the adaptive CD8+ T cell response during early life [22]. Notably, shifts we observed in the epitope dominance hierarchy during early life are temporally correlated with the changes we describe here in dendritic cells. The CD8+ T cell response in early life is characterized by a codominant response to two RSV epitopes, K^d^M2_82–90_ and D^b^M_187–195_. For infections that occur between day 10 and two weeks of age, CD8+ T cell responses become progressively K^d^M2_82–90_-skewed. In adults, the K^d^M2_82–90_ response is predictably 5–10 times higher than the response to D^b^M_187–195_ [22]. As CD103+ DCs play a major role in CD8+ T cell induction, we asked whether adult and neonatal CD103+ DCs differentially induced responses to these two epitopes. CD103+ DCs were sorted out of the MLN of mice one day after infection at 7 days old or as an adult, and co-cultured in a 1∶10 ratio with CFSE-labeled K^d^M2_82–90_ or D^b^M_187–195_-specific T cells isolated from adult TCR Tg mice without the addition of any exogenous peptide (Figure 5). The proliferation of TCR Tg T cells was measured by CFSE dilution three days later, and the percent of each original population that divided was calculated using the proliferation platform in FlowJo. Adult CD103+ DCs stimulated 52% of K^d^M2_82–90_-specific T cells to divide, while only 18% of D^b^M_187–195_-specific cells proliferated following co-culture with this same subset of sorted cells. These data align well with the described K^d^M2_82–90_-dominated response in adult mice. Following a three day co-culture with neonatal CD103+ DCs, 16% of K^d^M2_82–90_ specific cells and 12% of D^b^M_187–195_-specific cells were induced to proliferate (Figure 5A). This pattern of stimulation reflects the known codominance of these two epitope-specific populations following RSV infection of neonatal mice [22]. Data comparing the ratio of proliferation of K^d^M2_82–90_-specific cells/proliferation of D^b^M_187–195_-specific cells induced by CD103+ DCs sorted from neonates or adults 1 day post-infection from four independent experiments showed a significant difference in epitope-specific proliferation by neonatal and adult DCs (p = 0.0005, Figure 5B). As negative and positive controls, TCR Tg cells co-cultured in a 1∶1 ratio with splenocytes did not divide, and those co-cultured with splenocytes pulsed with 10^−6^M of specific peptide uniformly demonstrated near-100% proliferation (Figure 5).

### Limiting CD28-mediated costimulatory signals differentially affects epitope-specific CD8+ T cell responses

**Figure 6:**
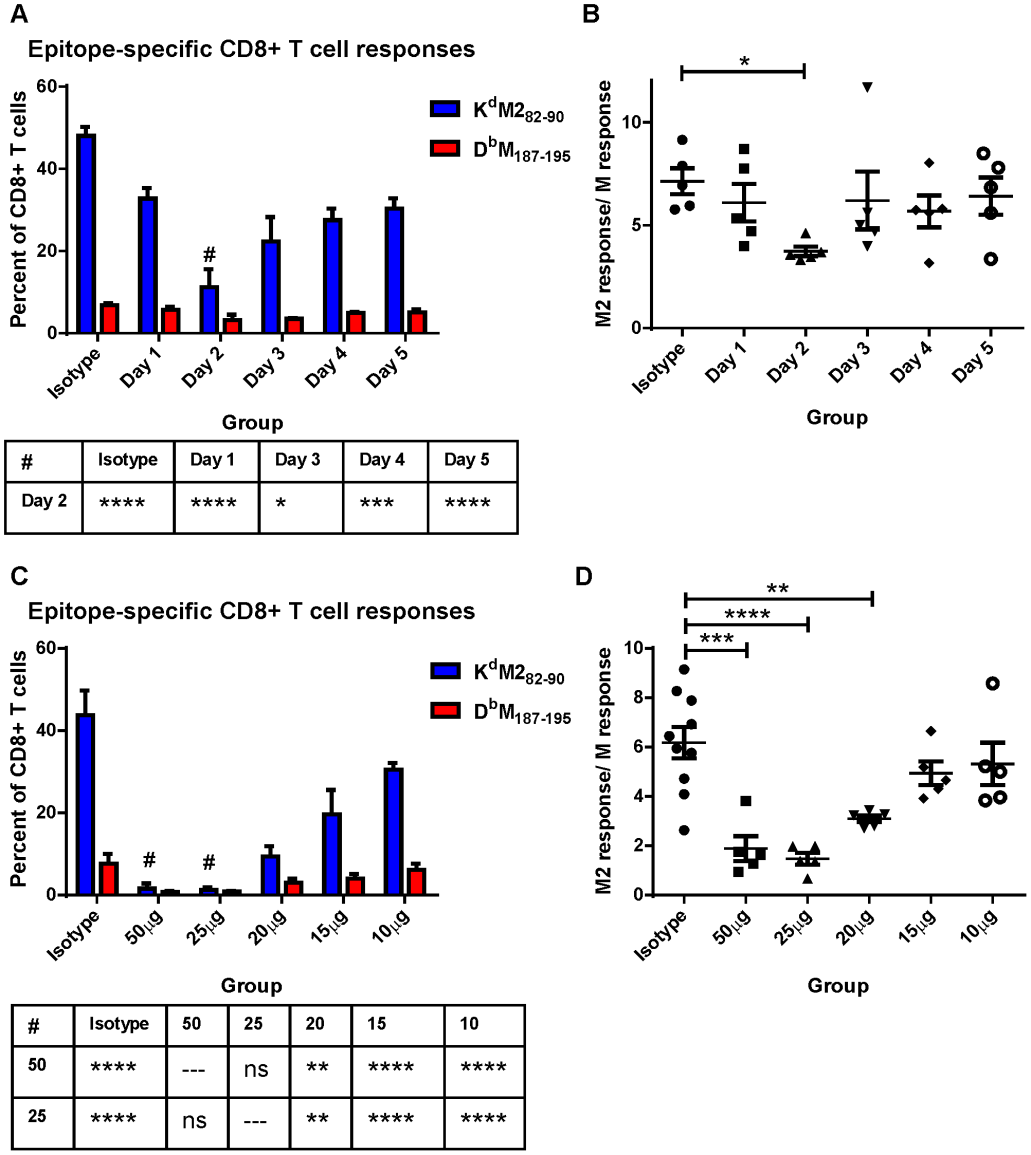
Modulating CD28-mediated costimulatory signals differentially affects K^d^M2_82–90_ and D^b^M_187–195_-specific responses. A) Mice were infected with RSV on day 0 and subsequently given IP injections of 20 µg each of antibodies against CD80 and CD86 at the indicated day post-infection. Epitope-specific CD8+ T cell responses were measured by surface and tetramer staining at 7 days post-infection. All groups were compared using a two-way ANOVA and Tukey's multiple comparisons tests, and there were no significant differences in the D^b^M_187–195_ response. Differences between the K^d^M2_82–90_ response on Day 2 and other groups are indicated below the figure (* p≤0.05, *** p≤0.001, **** p≤0.0001). B) The response ratio/immunodominance profile of CD8+ T cell responses was obtained for each mouse by dividing the K^d^M2_82–90_ response by the response to D^b^M_187–195_. * indicates p≤0.05 following one-way ANOVA and Dunnett's multiple comparisons test. C) Day 7 CD8+ T cell responses of mice injected with the indicated dose of anti-CD80 and CD86 at day 2 post-infection or isotype control antibodies (either 50 µg or 20 µg). All groups were compared using a two-way ANOVA and Tukey's multiple comparisons tests. Differences in the K^d^M2_82–90_ response between the 50 and 25 µg groups and all other groups are indicated below the figure (ns not significant, ** p≤0.01, and **** p≤0.0001). The D^b^M_187–195_ responses are significantly different between both the 50 and the 25 µg groups and isotype (p≤0.01) only. D) CD8+ T cell response ratios of mice treated with each dose of anti-CD80 and CD86 antibodies. Groups were compared with a one-way ANOVA and Tukey's multiple comparisons test (** p≤0.01, *** p≤0.001, **** p≤0.0001), and all error bars represent the SEM.

We hypothesized that differential induction of CD8+ T cell responses by neonatal and adult CD103+ DCs may be a result of differences we observed in the expression of both CD80 and CD86 costimulatory ligands on CD103+ DCs in the MLN. We asked how dampening CD28-mediated costimulation affected the CD8+ T cell response of adults using IP injection of antibodies that block the interactions of CD80 and CD86 with CD28 (16-10A and GL-1, respectively). RSV-infected mice were given a single dose containing 20 µg (approximately 0.9 mg/kg) of each antibody at 1, 2, 3, 4, or 5 days post-infection and CD8+ T cell responses were measured at day 7 and compared to mice injected with isotype control antibodies. CD80/CD86 antibodies had the largest effect on CD8+ T cell responses when administered two days post-infection (Figure 6A). In addition to dampening the overall CD8+ T cell response on day 2, limiting costimulation through CD28 differentially affected the K^d^M2_82–90_- and D^b^M_187–195_-specific responses. The K^d^M2_82–90_-specific response was reduced by 4-fold (mean of 48% to 11.2%), while the D^b^M_187–195_-specific response was only halved (6.8% to 3.2%). This differential effect lowered the K^d^M2_82–90_/D^b^M_187–195_ ratio in mice that received 80/86 treatment at two days post-infection (Figure 6B). We focused on day 2, and performed a dose-response by administering 50, 25, 20, 15, or10 µg of anti-CD80 and CD86. Administration of a single dose of 50 µg or 25 µg of anti-costimulatory antibody on day 2 nearly abrogated the RSV-specific CD8+ T cell response while doses between 20 and 10 µg of antibody had an intermediate effect (Figure 6C). Again, limiting CD28-mediated signaling demonstrated a differential effect on epitope-specific CD8+ T cells, significantly changing the K^d^M2_82–90_/D^b^M_187–195_ ratio in the 50, 25, and 20 µg dose groups (Figure 6D). Limiting CD28-mediated signaling in mice infected at 7 days of age had a similar effect, both dampening epitope-specific CD8+ T cell responses and significantly lowering the K^d^M2_82–90_/D^b^M_187–195_ ratio in pups treated with 7.5 to 10 µg of each antibody (Figure S8).

## Discussion

Here, we describe an evolution in the composition and function of the lung-migratory dendritic cell response following RSV infection in early life. We have defined both quantitative and qualitative defects in neonatal and early-life lung-migratory dendritic cell populations that contribute to attenuated and differential CD8+ T cell responses. While numerically similar to adult CD103+ DCs, neonatal and early life CD103+ DCs are less effective in the uptake and processing of soluble antigen, and express lower levels of costimulatory molecules. CD103+ DCs in neonates are also characterized by lower expression of their identifying marker, CD103. CD11b+ DCs appear in the MLN in dramatically lower numbers during early life, and demonstrate similar deficiencies in the uptake and processing of soluble antigens. We found that the DC response to respiratory infection changed rapidly during early life, indicating that even a one or two day difference in exposure to respiratory pathogens may dramatically change the resulting immune response.

It is no surprise that neonatal mice generate a lower CD8+ T cell response following RSV infection which may be due in part to deficiencies in early life innate immunity [22], [23]. In addition to a lower overall CD8+ T cell response, we previously reported that neonates generated a strikingly different immunodominance profile than adults following infection [22]. Here, we demonstrate that CD103+ DCs sorted from infected mice can induce CD8+ T cell responses in an age-dependent manner. Our results using TCR Tg cells as reporters of the ability of DCs to induce CD8+ T cell responses demonstrate that differences between neonates and adults are epitope-dependent, with some epitopes being greatly affected and others showing little age-dependent difference, and caution against using responses to a single epitope to generalize about DC function. The pattern of early CD8+ T cell induction by neonatal CD103+ DCs mirrors the immunodominance profile we have previously described in neonatal mice. Conversely, adult CD103+ DCs more proficiently stimulate the K^d^M2_82–90_ response that dominates the CD8+ T cell response in adults [22]. This age-dependent disparity in CD8+ T cell induction indicates that responses to particular epitopes may be favored over others during early life and implies a fundamental difference in the way in which neonatal CD103+ DCs process and present antigen that we are currently exploring further.

Due to reduced expression of both CD86 and CD80, neonatal CD103+ DCs in particular offer less costimulatory support to the adaptive response. Stimulation through CD28 is involved in the very early stages of T cell activation and is imperative for the elicitation of T cell responses [24], [25]. The importance of this interaction in the early generation of the adaptive response is consistent with our data indicating a maximal effect on CD8+ T cell responses when moderating these signals at 2 days post-infection. CD80/CD86-CD28 signaling is known to increase proliferation, antigen sensitivity, and survival following TCR/CD3 engagement [26]. While this costimulatory signal is also a critical determinant of CD4+ T cell function which may have an indirect effect on CD8+ T cell responses, a recent study has demonstrated that direct costimulation of CD8+ T cells is important for adequate priming and activation of the influenza-specific CD8+ T cell response [27]. This study suggested that CD28 costimulation of CD8+ T cells was more important than “help” from CD4+ T cells during influenza virus infection. Stimulation of CD8+ T cells through CD28 and other co-stimulatory pathways may be particularly critical in early life, when diminished CD4+ T cell responses are common. With regard to the CD4+ T cell response itself, other studies have demonstrated that strong costimulation through CD28 can suppress the induction of regulatory T cells and down-regulate Th17 development, indicating that different levels of CD28-mediated signaling may impact the differentiation of the CD4+ T cell response [28], [29]. We are further exploring the possibility that early life differences in the costimulatory capacity of CD103+ and CD11b+ DC populations are a determinant of CD4+ T cell differentiation and function.

CD27-CD70-mediated signaling has been found to promote the late expansion of NP-specific CD8+ T cell responses following influenza infection [30] and to bolster low affinity responses to help maintain a clonally diverse CD8+ T cell response [31]. When expression of CD80 and CD86 are low, CD28-mediated costimulation may impact immunodominance through a similar mechanism during early CD8+ T cell activation. Our previous work has shown that the polyclonal D^b^M_187–195_-specific response is of higher avidity than the K^d^M2_82–90_-specific response in RSV infected adults and neonates [22]. Early-life limitations of CD28-mediated costimulation may favor the induction of CD8+ T cells with the highest avidity for antigen and as a result, skew the response toward particular epitopes. Our work is consistent with older studies that used altered peptide ligands and TCR transgenic cells of a single specificity to demonstrate that the strength of the TCR-pMHC interaction determined the requirements for CD28-mediated costimulatory signals and indicated that weaker APC-T cell interactions have a greater dependence on co-stimulation for proliferation [32]. Although another previous study using soluble CD152 (CTLA4.Fc) to block CD80/CD86 interaction with CD28 indicated that this interaction does not appear to grossly influence immunodominance following influenza/PR8 infection [33], the CD80/86 interaction with CD28 appears to influence the pattern of responses in the RSV model. It is likely that co-stimulation is not the only factor that influences age-dependent differences in the CD8+ T cell response to RSV. While administration of sufficient blocking antibody to nearly eliminate the CD8+ T cell response caused a significant decrease in the K^d^M2_82–90_/D^b^M_187–195_ ratio, the resulting ratio of 2 does not fully recapitulate the reproducible ratio of 1 we find following neonatal infection with RSV [22].

RSV is a specialized early life pathogen in humans. The great success of this virus depends, in part, on the inability of infants to generate an effective immune response. CD8+ T cell responses are critical mediators of viral clearance following infection, and have been found to peak at convalescence in RSV-infected infants [34], [35]. The major goal of early life vaccination efforts to combat the toll of RSV-mediated morbidity and mortality is the generation of both effective T cell and antibody responses in this vulnerable population. A better understanding of the mechanisms underlying the shortcomings of the early life immune response will provide targets for the elicitation of optimal immune responses in neonates. Our data suggest that the CD80/CD86-CD28 axis may be exploited in the design of pediatric vaccines to promote the generation of more “adult-like” adaptive responses.

## Materials and Methods

### Ethics statement

All mice used in these studies were maintained according to the guidelines of the NIH Guide to the Care and Use of Laboratory Animals and experimental procedures had the approval of the Animal Care and Use Committee of the Vaccine Research Center (VRC), National Institute of Allergy and Infectious Diseases at the National Institute of Health under protocol numbers 10-315 and 11-345. All mice were housed in a facility fully accredited by the Association for Assessment and Accreditation of Laboratory Animal Care International (AAALAC). Animal procedures were conducted in strict accordance with all relevant federal and National Institutes of Health guidelines and regulations.

### Mice, RSV infections and other treatments

BALB/c (female) and C57BL/6 (male) breeders were purchased from Jackson Labs and CB6F1 pups were obtained by time-mating. Adult (8–14 weeks old) female CB6F1/J mice (Jackson Labs, Bar Harbor, ME or bred in-house) were used. TCR transgenic mice were created using TCR CDR3 sequences selected based on single-cells sequencing of the K^d^M2_82–90_ and D^b^M_187–195_-specific CD8+ T cell responses [36].TCR β chain cDNA (full-length, fully recombined) sequence was cloned into a transgenic vector that exploits the H-2Kb promoter and an Ig enhancer, and a paring TCR α chain cDNA sequence was cloned into a CD2-based expression vector as previously described [37]. K^d^M2_82–90_-specific transgenic T cells are TRBV13-2*01 (www.imgt.org) with the CDR3 sequence CASGAGTGYAEQFF, and TRAV7-3*01 with the CDR3 sequence CAVNSGYNKLTF on the BALB/c background. D^b^M_187–195_-specific transgenic T cells are TRBV17*01 with the CDR3 sequence of CASSDWGGYEQYF and TRAV7-5*01 with the CDR3 sequence of CAVRGSSGNKLIF on a C57BL/6 background. All TCR trangenics were produced by NCI-Frederick Laboratory Animal Sciences Program (LASP) transgenic mouse model service and subsequently bred as heterozygotes in-house. All mice were housed in our animal care facility at NIAID under specific, pathogen-free conditions, and maintained on standard rodent chow and water supplied *ad libitum*. All studies were reviewed and approved by the NIH Animal Care and Use Committee. Mice were anesthetized using isoflurane (3%), and mice of all ages were infected intranasally with 2×10^6^ PFU live RSV in 10% EMEM (100 µl for adults, 15–50 µl for younger mice depending on mouse age and size). In some cases, mice were infected with RSV that expresses GFP following infection (rgRSV) previously described [38]. Neonatal mice were infected at day 7 of life unless stated otherwise. All mice were euthanized by lethal injection with pentobarbital (250 mg/kg). Experiments involving co-administration either OVA-FITC or OVA-DQ (invitrogen) were performed by adding either 50 µg/mouse directly to virus preparations prior to intranasal administration under isoflurane anesthesia. Antibodies to anti-CD80 (16-10A1) and anti-CD86 (GL-1) and corresponding isotype control antibodies (2A3 and polyclonal IgG) were purchased from Bio X Cell (West Lebanon, NH) and a dose of 20 µg of each were injected IP into RSV-infected animals in PBS in a total volume of 200 µL/mouse to determine the optimal day for treatment. Subsequent dose response studies were performed using between 10 and 50 µg of each antibody in adult mice and between 5 and 10 µg in neonates.

### Flow cytometry

Mice were sacrificed and lung and/or MLN tissues were harvested at the indicated times post-infection. In most cases, tissues from 3–7 mice were pooled to generate one sample. Lung tissues were disrupted by tissue dissociation using a GentleMACS machine (Miltenyi). Mononuclear cells were purified using Fico-LITE at room temperature, washed, then resuspended in 10% RPMI. MLN tissues were ground between the ends of two frosted glass slides and mononuclear cells were isolated as described above. After isolation, cell preparations were stained with fluorochrome-labeled antibodies to the following cell surface markers: CD11c (N418), CD11b (M1/70), CD103 (2E7), PDCA (clone), CD8α (53-6.7), MHC class II (M5/114.15.2), CD3 (145-2C11) purchased from BD biosciences, eBioscience, Miltenyi or Biolegend at concentrations predetermined by antibody titration. All staining was done for 20 minutes at 4°C in FACS staining buffer (PBS + 1% FBS + 0.05% sodium azide). Samples were collected on an LSR-II flow cytometer (BD, San Jose, CA) and data were analyzed using FlowJo version 9.9.10, the gating strategies for lung and MLN are presented in Figures S1 and S2, respectively.

### Dendritic cell: T cell co-culture

DC samples for DC:T coculture were stained as above except MACS staining buffer without azide was used, and samples were sorted on a FACSAria into R-10. CD8+ T cells were isolated from splenocytes of TCR transgenic mice with T cells specific for either RSV-K^d^M2_82–90_ or RSV-D^b^M_187–195_ using a CD8α T cell isolation kit (Miltenyi), then labeled with 5 µM CFSE for 5 minutes at room temperature followed by three washes with FBS containing media. CD8+ T cells of each specificity (100,000) were co-cultured with FACSAria-sorted populations of CD103+ DCs (10,000) from mice infected at 7 days of age or as an adult without the addition of exogenous peptide. CD8+ T cells were incubated with splenocytes pulsed with 10^−6^M cognate peptide as a positive control, or splenocytes with no peptide as a negative control. Three days later, all samples were harvested and stained with antibodies to CD8, CD3, and with ViViD (for viability) prior to collection on a LSRII.

### Statistical analysis

Statistical analyses were performed using GraphPad prism version 6.00 for Windows, GraphPad Software, LaJolla California USA, www.graphpad.com using either a student's t-test, or one-way or two-way ANOVA with the indicated post-tests for multiple comparisons.

## Supporting Information

Figure S1 **Gating strategy for CD103+ and CD11b+ dendritic cell populations in the lung.** CD103+ and CD11b+ populations were gated from the live ClassII high, CD11c+ population after gating for both singlets and live (aqua-excluding) cells.
(TIFF)

Figure S2 **Gating of GFP+ CD103+ DCs in the MLN following rgRSV infection.** GFP positive CD103+ DCs were gated by comparison to naïve and wtRSV infected mice.
(TIF)

Figure S3 **MLN identification and gating strategy for CD103+ and CD11b+ dendritic cell populations in the MLN.** A) Photographs of the posterior MLN in neonates or adults 3 days after infection with RSV. The red arrow indicates the MLN, and the black arrow indicates the thymus. B) Gating strategy for CD103+ and CD11b+ DCS in the MLN. The population gated is indicated above each plot. The CD103+ and CD11b+ DCs are gated from the ClassIIhi population.
(TIFF)

Figure S4 **Age-dependent changes in the composition of CD103+ and CD11b+ DCs in the MLN.** Gating of CD103+ and CD11b+ DCs from the MHC ClassIIhi, CD11c+ population in the MLN three days after infection of mice at the indicated ages.
(TIF)

Figure S5 **Composition of CD103+ and CD11b+ DCs in the MLN of 7 day old or adult naïve CB6F1 mice. A)** MLN were harvested from 7 day old or adult mice (8 lymph nodes/sample) and stained as indicated in the materials and methods and Figure S3. Samples were run to completion on the flow cytometer, and the number of CD103+ and CD11b+ DCs isolated per mouse were calculated. *** p≤0.001 following two-way ANOVA and Sidak's multiple comparisions test. B) The CD103/CD11b+ DC ratio in naïve neonatal or adult mice. Error bars represent the SEM, and the groups were analyzed by student's t-test.
(TIFF)

Figure S6 **Representative gating of CD103+ DCs in the MLN following co-administration of ova-FITC or ova-DQ.** A) ova-FITC positive CD103+ DCs were identified in the MLN of mice of different ages one day after infection with RSV and co-administered ova-FITC compared to control (RSV infection only). B) ova-DQ positive CD103+ DCs one day after RSV infection and ova-DQ co-administration in mice of different ages.
(TIFF)

Figure S7 **Representative data showing expression of the costimulatory molecules CD86, CD80, and CD70 on CD103+ and CD11b+ DCs in the MLN three days after infection of mice at different ages.** Data plots showing the age-dependent expression level of costimulatory molecules on gated populations of CD103+ and CD11b+ DCs in the MLN three days after infection of mice at different ages.
(TIFF)

Figure S8 **Modulating CD28-mediated costimulatory signals differentially affects K^d^M2_82–90_ and D^b^M_187–195_-specific responses in neonatal CB6F1 mice.** A) Neonatal mice were infected with RSV at 7 days old. Two days post infection, they were given either isotype antibodies (10 µg) or 10, 7.5, or 5 µg each of antibodies against CD80 and CD86 IP. Epitope-specific CD8+ T cell responses were measured by surface tetramer staining 7 days post-infection. B) CD8+ T cell response ratios of mice treated with varying doses of anti-CD80 and CD86 antibodies. Groups were compared with a one-way ANOVA and Tukey's multiple comparisons test (** p≤0.01, *** p≤0.001, **** p≤0.0001), and all error bars represent the SEM.
(TIFF)
