# Supplementary figures and images for: Quantitative and Qualitative Deficits in Neonatal Lung-Migratory Dendritic Cells Impact the Generation of the CD8+ T Cell Response
Source: PLoS Pathog. 2014 Feb 13;10(2):e1003934. doi: 10.1371/journal.ppat.1003934 (PMC3923758; doi:10.1371/journal.ppat.1003934)

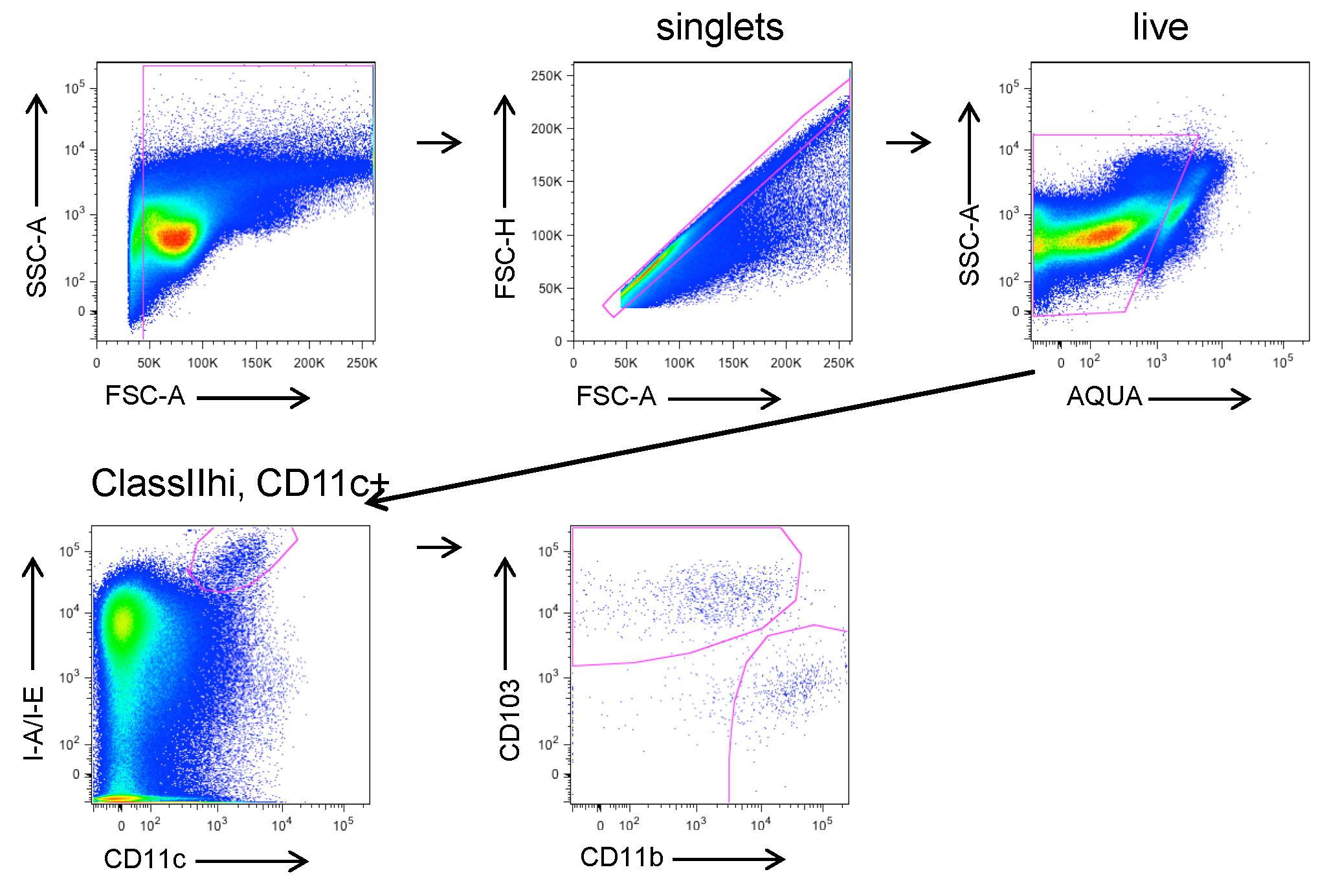

Supplement: Figure S1 — Gating strategy for CD103+ and CD11b+ dendritic cell populations in the lung. CD103+ and CD11b+ populations were gated from the live ClassII high, CD11c+ population after gating for both singlets and live (aqua-excluding) cells. (TIFF) [file ppat.1003934.s001.tiff]

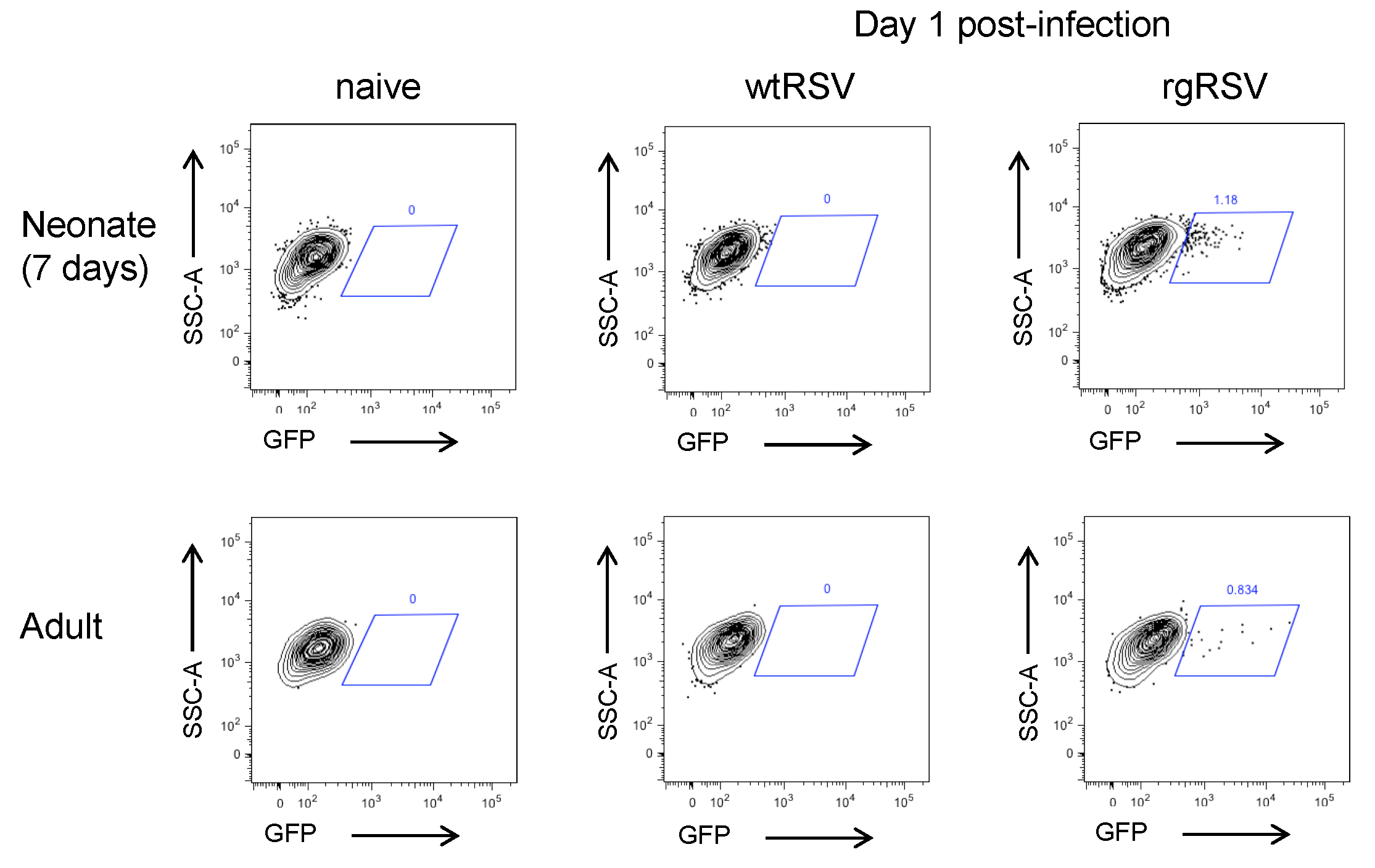

Supplement: Figure S2 — Gating of GFP+ CD103+ DCs in the MLN following rgRSV infection. GFP positive CD103+ DCs were gated by comparison to naïve and wtRSV infected mice. (TIF) [file ppat.1003934.s002.tif]

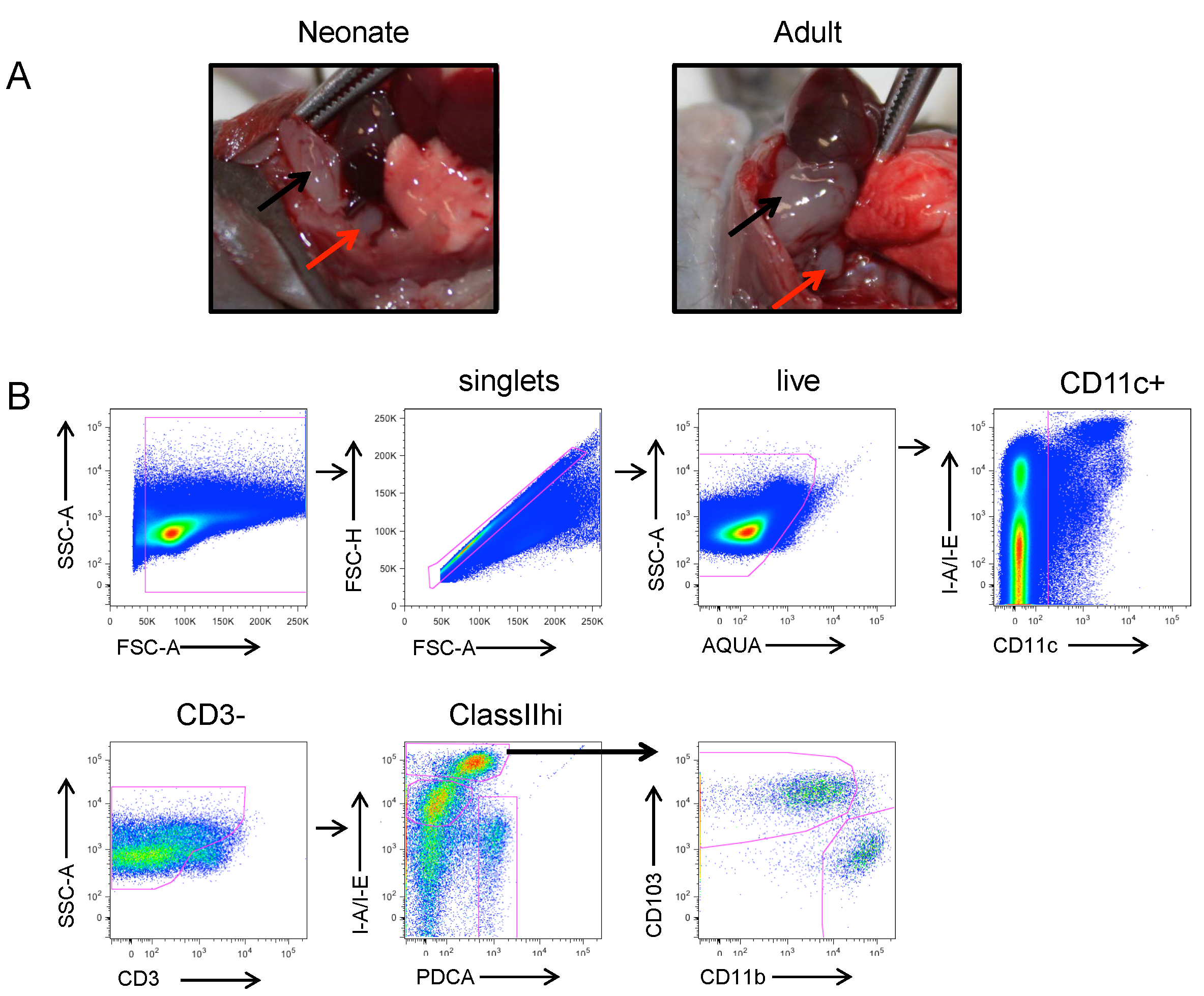

Supplement: Figure S3 — MLN identification and gating strategy for CD103+ and CD11b+ dendritic cell populations in the MLN. A) Photographs of the posterior MLN in neonates or adults 3 days after infection with RSV. The red arrow indicates the MLN, and the black arrow indicates the thymus. B) Gating strategy for CD103+ and CD11b+ DCS in the MLN. The population gated is indicated above each plot. The CD103+ and CD11b+ DCs are gated from the ClassIIhi population. (TIFF) [file ppat.1003934.s003.tiff]

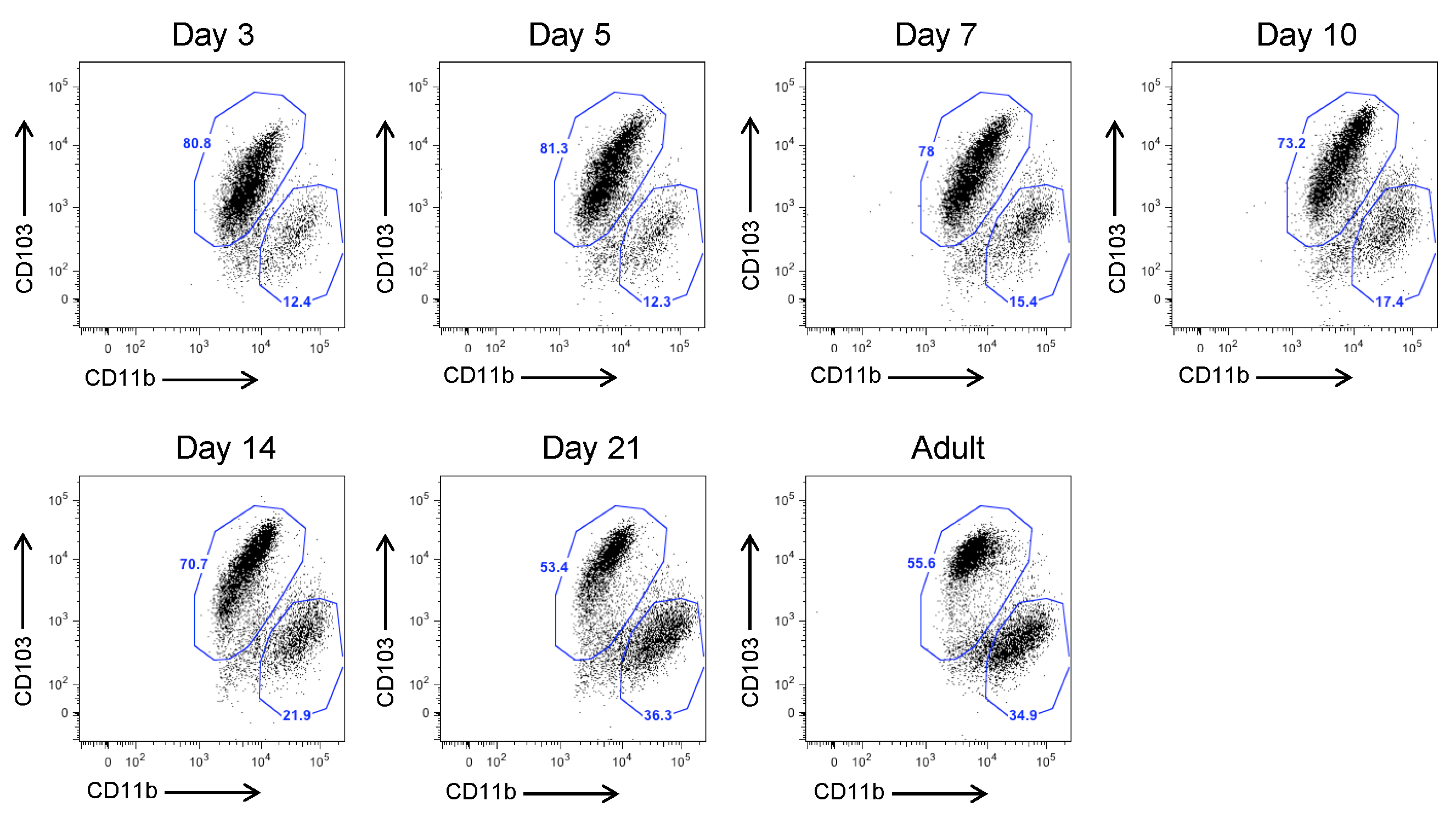

Supplement: Figure S4 — Age-dependent changes in the composition of CD103+ and CD11b+ DCs in the MLN. Gating of CD103+ and CD11b+ DCs from the MHC ClassIIhi, CD11c+ population in the MLN three days after infection of mice at the indicated ages. (TIF) [file ppat.1003934.s004.tif]

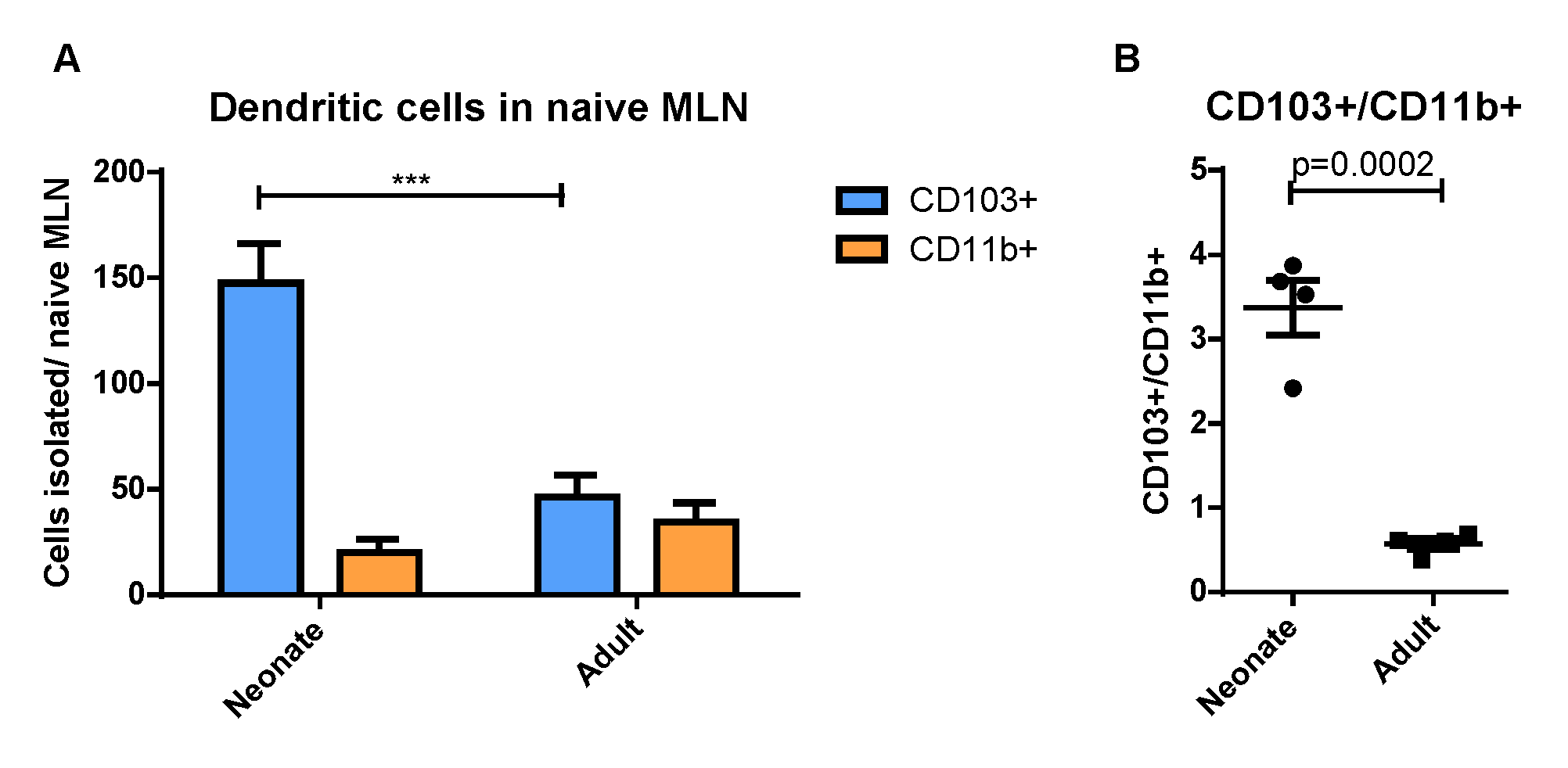

Supplement: Figure S5 — Composition of CD103+ and CD11b+ DCs in the MLN of 7 day old or adult naïve CB6F1 mice. A) MLN were harvested from 7 day old or adult mice (8 lymph nodes/sample) and stained as indicated in the materials and methods and Figure S3. Samples were run to completion on the flow cytometer, and the number of CD103+ and CD11b+ DCs isolated per mouse were calculated. *** p≤0.001 following two-way ANOVA and Sidak's multiple comparisions test. B) The CD103/CD11b+ DC ratio in naïve neonatal or adult mice. Error bars represent the SEM, and the groups were analyzed by student's t-test. (TIFF) [file ppat.1003934.s005.tiff]

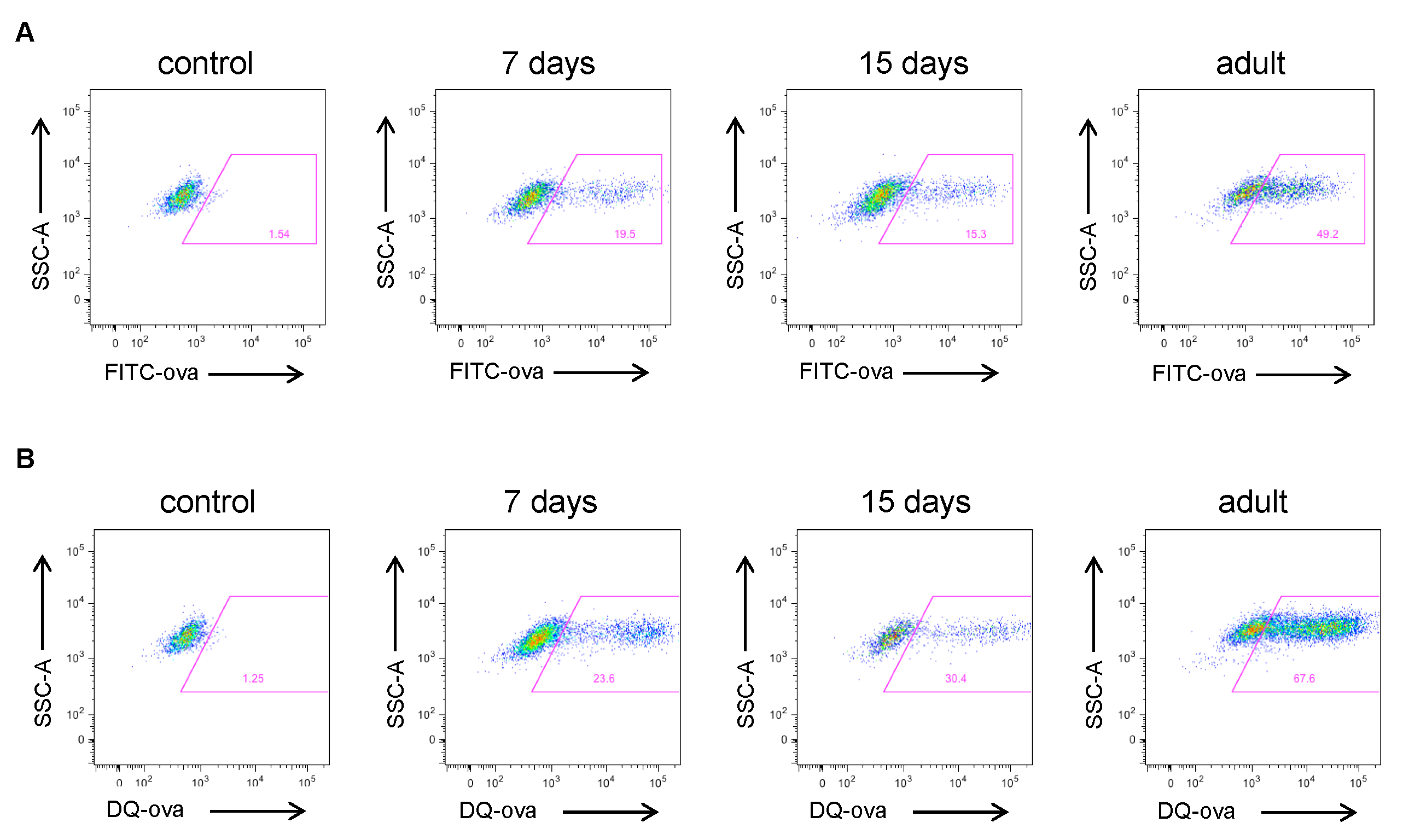

Supplement: Figure S6 — Representative gating of CD103+ DCs in the MLN following co-administration of ova-FITC or ova-DQ. A) ova-FITC positive CD103+ DCs were identified in the MLN of mice of different ages one day after infection with RSV and co-administered ova-FITC compared to control (RSV infection only). B) ova-DQ positive CD103+ DCs one day after RSV infection and ova-DQ co-administration in mice of different ages. (TIFF) [file ppat.1003934.s006.tiff]

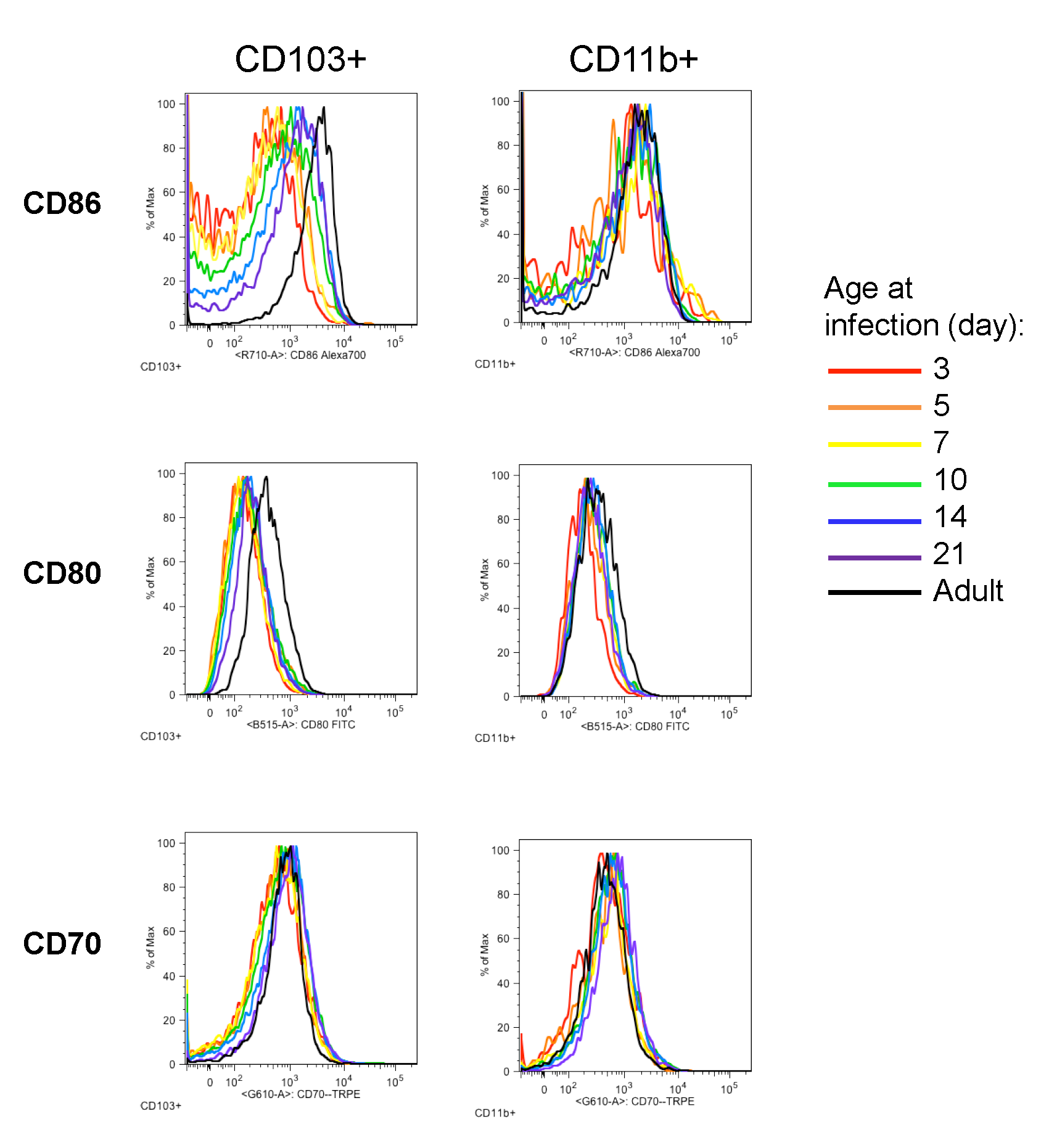

Supplement: Figure S7 — Representative data showing expression of the costimulatory molecules CD86, CD80, and CD70 on CD103+ and CD11b+ DCs in the MLN three days after infection of mice at different ages. Data plots showing the age-dependent expression level of costimulatory molecules on gated populations of CD103+ and CD11b+ DCs in the MLN three days after infection of mice at different ages. (TIFF) [file ppat.1003934.s007.tiff]

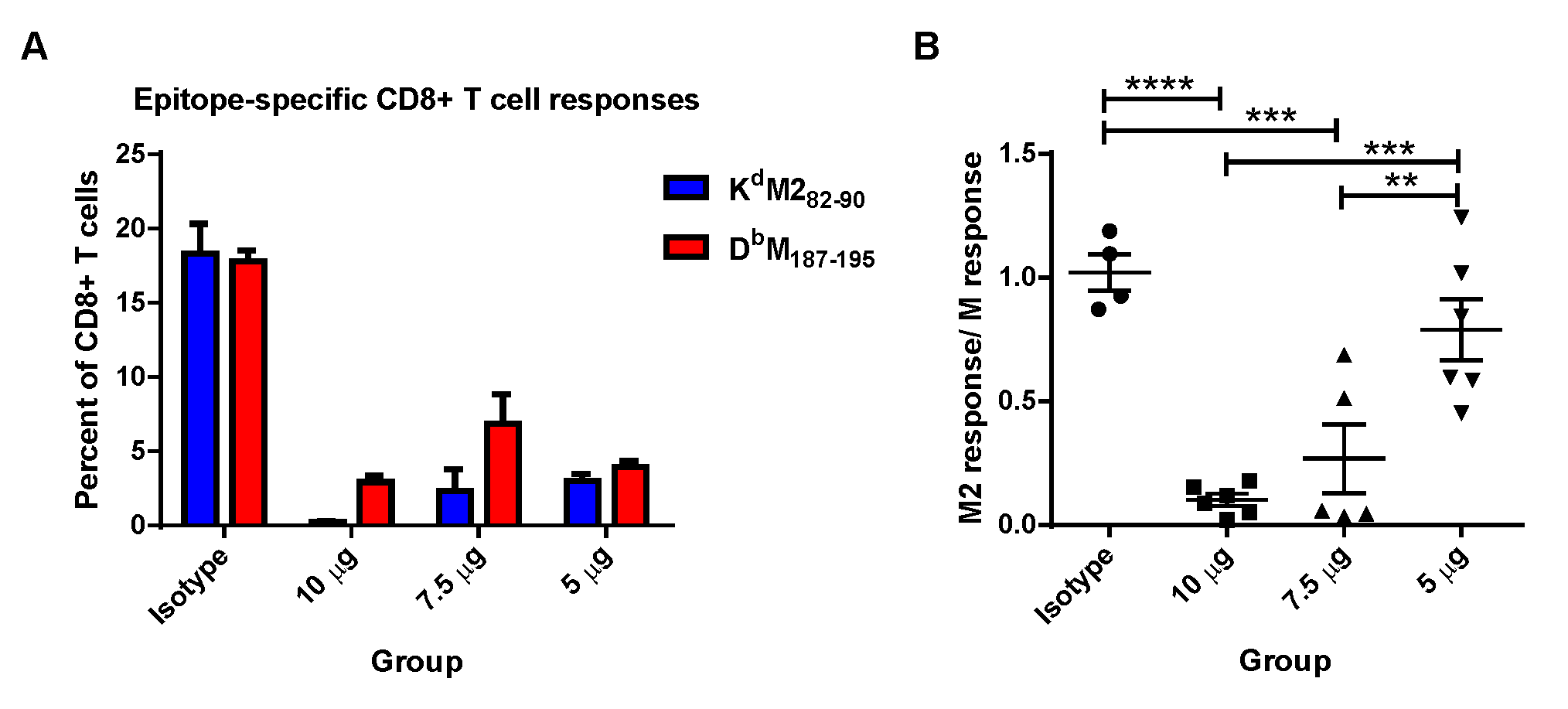

Supplement: Figure S8 — Modulating CD28-mediated costimulatory signals differentially affects KdM282–90 and DbM187–195-specific responses in neonatal CB6F1 mice. A) Neonatal mice were infected with RSV at 7 days old. Two days post infection, they were given either isotype antibodies (10 µg) or 10, 7.5, or 5 µg each of antibodies against CD80 and CD86 IP. Epitope-specific CD8+ T cell responses were measured by surface tetramer staining 7 days post-infection. B) CD8+ T cell response ratios of mice treated with varying doses of anti-CD80 and CD86 antibodies. Groups were compared with a one-way ANOVA and Tukey's multiple comparisons test (** p≤0.01, *** p≤0.001, **** p≤0.0001), and all error bars represent the SEM. (TIFF) [file ppat.1003934.s008.tiff]
